# Evidence for a common mechanism for spontaneous rhythmic contraction and myogenic contraction induced by quick stretch in detrusor smooth muscle

**DOI:** 10.1002/phy2.168

**Published:** 2013-11-22

**Authors:** S Omid Komari, Patrick C Headley, Adam P Klausner, Paul H Ratz, John E Speich

**Affiliations:** 1 Department of Mechanical and Nuclear Engineering, Virginia Commonwealth UniversityRichmond, Virginia, 23284; 2 Department of Biomedical Engineering, Virginia Commonwealth UniversityRichmond, Virginia, 23284; 3 Department of Surgery, Virginia Commonwealth UniversityRichmond, Virginia, 23298; 4 Departments of Biochemistry & Molecular Biology and Pediatrics, Virginia Commonwealth UniversityRichmond, Virginia, 23298

**Keywords:** Autonomous contraction, bladder, muscle twitch, phasic contraction, smooth muscle mechanics

## Abstract

Detrusor smooth muscle exhibits myogenic contraction in response to a quick stretch (QS) as well as spontaneous rhythmic contraction (SRC); however, whether the same population of actomyosin crossbridges with a common regulatory mechanism is responsible for these two types of contraction has not been determined. Detrusor strips from New Zealand white rabbit bladders were allowed to develop SRC at a reference muscle length (*L*_ref_), or rhythmic contraction (RC) was induced with tetraethylammonium (TEA). Multiple 10-msec stretches of 15% *L*_ref_ were then imposed at *L*_ref_ randomly during the rhythm cycle, and the nadir-to-peak (NTP) tension amplitude of the resulting myogenic contraction was measured. The amplitude and period of the rhythm cycle were measured prior to each QS. NTP was larger when a QS was imposed during a portion the cycle when tension was smaller (*n* = 3 each SRC and TEA-induced RC). These data suggest that when the rhythmic mechanism was mostly inactive and tension was near a minimum, a larger portion of a shared population of crossbridges was available to produce a myogenic response to a QS. Rho kinase, cyclooxygenase-1, and cyclooxygenase-2 inhibitors (H-1152, SC-560, and NS-398) affected SRC amplitude and NTP amplitude following a QS to the same degree (*n* = 3 each drug), providing additional evidence to support the hypothesis that a common mechanism is responsible for SRC and myogenic contraction due to QS. If a common mechanism exists, then QS is a potential mechanical probe to study SRC regulation and its alteration in overactive bladder.

## Introduction

Detrusor smooth muscle in the bladder can exhibit spontaneous nonvoiding contractions during the urine collection phase (Drake et al. 2005), including relatively low amplitude spontaneous rhythmic contraction (SRC). SRC has been identified in detrusor in a range of mammalian species, including rabbits (Shenfeld et al. 1999), rats (Kanai et al. 2007), and humans (Biers et al. 2006); and is also present in other muscles, including gastrointestinal (Huizinga et al. 2000) and vascular smooth muscle (Griffith 1996). SRC is elevated in detrusor strips from patients with overactive bladder (Kinder and Mundy 1987), and SRC is likely responsible for micromotion observed during filling of human bladders, which is also elevated in patients with overactive bladder (Drake et al. 2005).

When subjected to a quick stretch (QS) or hypo-osmotic solutions, human (Masters et al. 1999), rabbit (Burnstock and Prosser 1960), and mouse (Ji et al. 2002) detrusor can exhibit a phasic myogenic contraction independent of neural input. The myogenic contraction has been studied extensively in vascular smooth muscle, where it has an important role in blood flow regulation (Davis and Hill 1999), and rapid volume increases can produce myogenic bladder pressure transients (Andersson et al. 1988). In rabbit detrusor, there are several similarities between SRC and myogenic contractions. Both types of contraction have small amplitudes, with SRC producing ∼5–12% of peak active tension (Ratz and Miner 2003) and QS-induced myogenic contraction producing ∼4–13% of peak active tension (Poley et al. 2008). In addition, the amplitudes of both types of contraction are muscle length dependent, with SRC amplitude increasing at longer muscle lengths (Byrne et al. 2013) and the myogenic contraction amplitude increasing with the length of the QS (Poley et al. 2008). Both types of contraction are also phasic. In rabbit detrusor, SRC appears as a somewhat sinusoidal tension wave, or a combination of waves, with varying amplitude(s) and a frequency or frequencies ranging from ∼2 to 11 cycles/min (∼5–30 sec between peaks) (Byrne et al. 2013). The myogenic response to QS exhibits a refractory period of 10–30 sec, with a QS repeated after 10 sec provoking a significantly smaller contraction and a QS repeated after 30 sec provoking only a slightly smaller contraction (Poley et al. 2008). Thus, the refractory period between myogenic contractions due to QS and the timing of SRC are similar, suggesting a common oscillatory regulation system.

This study was designed to test the hypothesis that a common contractile mechanism is responsible for SRC and for the myogenic contractile response following a QS in detrusor. This hypothesis was tested using a mechanical protocol during which QSs were imposed randomly throughout the rhythm cycle to determine whether (1) the myogenic response would be relatively large when tension in the rhythm cycle was small, suggesting that the common mechanism was primarily “off” at that point in the rhythm cycle, leaving a substantial fraction that was “turned on” by the QS; and (2) the myogenic response would be relatively small at a point when tension in the rhythm cycle was large, suggesting that the common mechanism was mostly “on” and only a small fraction remained to be “turned on” by the QS. The hypothesis was also tested using a pharmacological protocol to determine whether Rho kinase (ROCK), cyclooxygenase-1 (COX-1), and cyclooxygenase-2 (COX-2) inhibitors (H-1152, SC-560, and NS-398, respectively) affected SRC and the myogenic contractile response due to QS to the same degree.

Uniform tissue contractions seen during SRC or a QS-induced contraction require cell-to-cell synchronization of contraction. Not every smooth muscle cell of a detrusor muscle bundle is innervated by a postganglionic parasympathetic fiber (Elbadawi 1995), suggesting that cell-to-cell coupling is necessary to synchronize contraction. Detrusor cells are poorly coupled electrically (Bramich and Brading 1996), and therefore, mechanical coupling may be responsible for synchronization. Elbadawi (1995) and Ji et al. (2002) have proposed a mechanical coupling model, in which rapid contraction of one muscle cell stimulates surrounding cells by rapidly stretching them to propagate contraction, including SRC, throughout a bundle of cells. This study will test the hypothesis that a common contractile mechanism is responsible for both SRC and QS-induced contraction, which is consistent with a model, in which cell stretch is a stimulus to propagate SRC throughout a bundle or bundles of detrusor cells.

## Methods

### Tissue preparation

All experiments involving rabbits were conducted within the appropriate animal welfare guidelines and regulations and were approved by the VCU Institutional Animal Care and Use Committee. Whole bladders were harvested from adult female New Zealand white rabbits (2–4 kg) sacrificed by anesthesia overdose. Bladders were washed, cleaned of adhering serosa and fat, and stored in cold (0–4°C) modified physiologic salt solution. Thin strips (∼0.2 mm thick) of longitudinal detrusor, without mucosa, were dissected from the bladder wall close to the dome by following the natural bundling, as described previously (Almasri et al. 2010a).

### Solutions and drugs

Modified physiological salt solution (PSS) was composed of NaCl, 140 mmol/L; KCl, 4.7 mmol/L; MgSO_4_, 1.2 mmol/L; CaCl_2_, 1.6 mmol/L; Na_2_HPO_4_, 1.2 mmol/L; morpholinopropanesulfonic acid, 2.0 mmol/L (adjusted to pH 7.4 at either 0 or 37°C, as appropriate); Na_2_ ethylenediamine tetraacetic acid, 0.02 mmol/L; and dextrose, 5.6 mmol/L. Modified PSS, in which 110 mmol/L KCl was substituted isosmotically for NaCl, (KPSS) was used to induce muscle contractions, and a Ca^2+^-free solution (0-Ca), composed of PSS without CaCl_2_, was used to abolish rapid crossbridge cycling for preload tension measurements (Shenfeld et al. 1999). ROCK inhibitor 0.3 *μ*mol/L H-1152 (Toronto Research Chemicals, Toronto, ON, Canada), COX-1 inhibitor 0.1 *μ*mol/L SC-560 (Cayman Chemical, San Antonio, TX), and COX-2 inhibitor 0.1 *μ*mol/L NS-398 (Cayman Chemical) were used to inhibit detrusor contractions (Teixeira et al. 2007; Collins et al. 2009). Drugs were dissolved in dimethyl sulfoxide (DMSO), which was added at a final concentration of 0.1%. At this percentage, the DMSO does not affect the myogenic response (Poley et al. 2008).

### Apparatus, muscle strip setup, and reference length determination

One end of each detrusor strip was clamped to a rigid postconnected to a micrometer for manual length adjustments, and the other end was clamped in a small aluminum foil clip which was connected to a computer-controlled lever (model 300H; Aurora Scientific, Aurora, ON, Canada) to record tension and to induce time-controlled changes in muscle length. Tension and length signals were digitized (PCI-6024E; National Instruments, Austin, TX) and stored electronically for analyses. Each strip was secured with a cold, zero-preload length of ∼4 mm and then equilibrated in aerated PSS at 37°C. The mechanical and pharmacological protocols in this study were performed based on a reference length (*L*_ref_) for each tissue. To determine *L*_ref_, the detrusor strips were equilibrated at 4 mm in PSS for 10 min, and then stretched to 5 mm for another 10 min. Then, tissues were stretched to a load of 0.5 g and permitted to stress relax for 30 min. Next, strips were stretched to a load of 1.0 g and permitted to isometrically stress relax for another 30 min. At the end of each experiment, tissues were contracted isometrically to determine the maximum KCl-induced tension (*T*_max_KCl_) at that muscle length which was subsequently used as *L*_ref_. To reduce tissue-to-tissue variability, tension produced by myogenic contraction or RC was reported as a fraction *T*_max_KCl_.

### QS protocol

**Figure 1:**
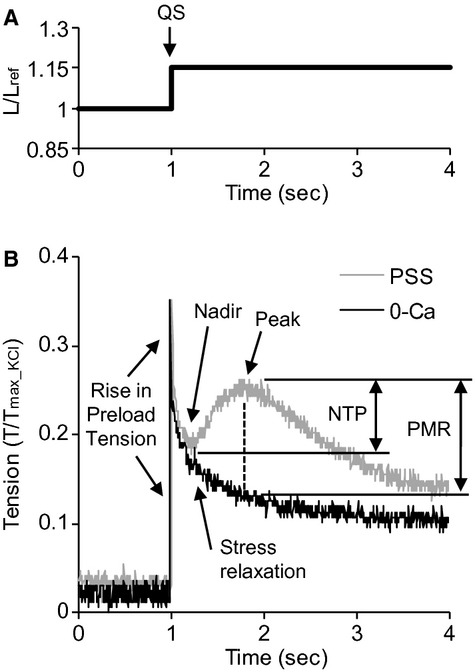
Quick stretch (QS) protocol from *L*_ref_ to 1.15 *L*_ref_ in 10 msec (A) and the corresponding rise in preload tension, subsequent stress relaxation, and subsequent myogenic contraction or lack of contraction from a detrusor strip incubated in physiological salt solution (PSS) or 0-Ca (B). Tension (T) was normalized to the maximum KCl-induced tension at 1.15 *L*_ref_ (*T*_max_KCl_). Peak myogenic response (PMR) tension was calculated as the difference between the 0-Ca and PSS curves at the peak of the myogenic contraction, and nadir-to-peak (NTP) tension was calculated as the difference between the nadir and subsequent peak of the PSS tension curve.

QSs were from *L*_ref_ to 115% *L*_ref_ with a stretch duration of 10 msec (Fig. 1A) as described previously (Poley et al. 2008). At the end of each QS, tissues were held isometrically at 115% *L*_ref_ for 10 sec and then returned to *L*_ref_. Tissues responded to QS with an immediate increase in tension followed by rapid stress relaxation (Fig. 1B). Following a brief latency period, tissues in PSS ceased to stress relax and produced a phasic myogenic contraction; however, in 0-Ca solution stress relaxation continued toward a steady-state tension value (Fig. 1B). Nadir-to-peak (NTP) tension of the myogenic contraction was measured from the minimum stress-relaxed value before the contraction to the peak of the contraction (Fig. 1B). The peak myogenic response (PMR) was calculated as the tension difference between the peak of the myogenic contraction and the stress-relaxed value in 0-Ca at the same time from the QS (Fig. 1B). The PMR tension induced by a QS has been shown to increase with increased stretch amplitude and increased stretch rate (Poley et al. 2008), and the stretch magnitude (15% *L*_ref_) and rate (10 msec) (Fig. 1A) were selected to induce a near maximal myogenic contraction.

### Quantification of RC

**Figure 2:**
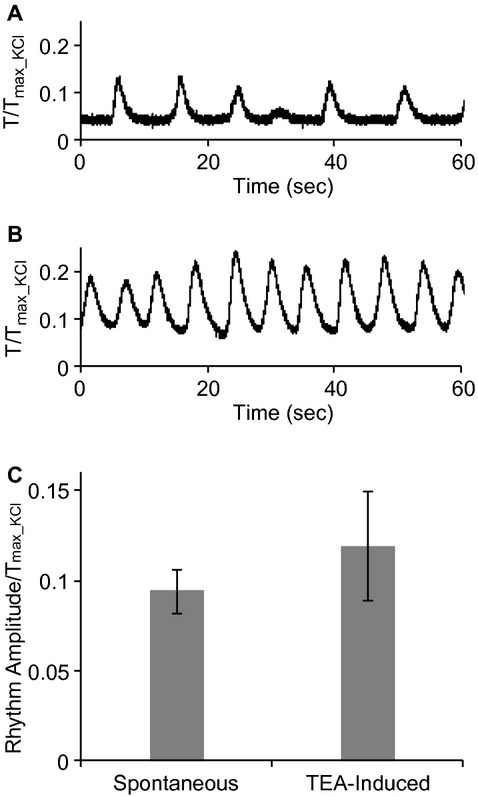
Examples of spontaneous (A) and 2 mmol/L tetraethylammonium (TEA)-induced (B) rhythmic contraction. (C) TEA-induced rhythmic amplitude was not significantly different from spontaneous rhythmic amplitude (*P* > 0.05*, n* = 3).

**Figure 3:**
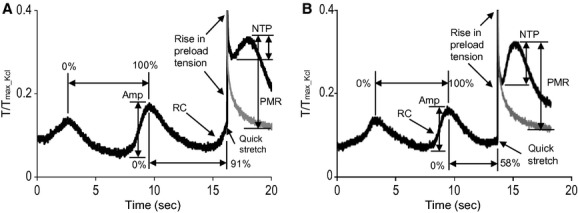
(A–B) Examples of how induction of a quick stretch (QS) at different time points during a tetraethylammonium (TEA)-induced rhythmic cycle affects the amplitude of the myogenic contraction. Amplitude (Amp) and duration from peak (0%) to peak (100%) of the rhythm cycle were measured immediately prior to each stretch. Peaks of sequential rhythmic contractions were at 0% and near 100%, and because of the asymmetric shape of the rhythmic train of contractions, the trough between two twitch contractions was ∼40–80% of the spontaneous rhythmic contraction (SRC) cycle. Myogenic contractions for stretches imposed at 91% (A) and 58% (B) of the rhythm cycle are shown. Tension (T) was normalized to the maximum KCl-induced tension at 1.15 *L*_ref_ (*T*_max___KC__l_). Nadir-to-peak (NTP) and peak myogenic response (PMR) tensions were calculated as described in Figure 1. Note that the peak of the rise in preload tension prior to the myogenic contraction has been cut off due to the tension scale (A, 0.53 and B, 0.51).

In one group of detrusor strips, SRC was allowed to spontaneously develop at *L*_ref_ (Fig. 2A). To increase the frequency of RCs in another group of tissues, the potassium channel blocker tetraethylammonium (TEA), which depolarizes the plasma membrane by reducing potassium conductance (Wellner and Isenberg 1994), was added at a concentration of 2 mmol/L (Fig. 2B). TEA was utilized because it typically induces RCs with a more consistent frequency (Fig. 2B). Tension amplitudes were measured for 19 or more cycles, and average tension amplitudes for SRC and 2 mmol/L TEA-induced transient contractions were not significantly different (Fig. 2C). Rhythm cycle duration was measured from tension peak (0%) to tension peak (100%) for the cycle (Fig. 3).

### QS and rhythm synchronization

To test the hypothesis that the myogenic contraction following QS would be relatively large (or relatively small) at a point in the rhythm cycle when tension was relatively small (or relatively large), multiple QSs (19–23 per strip) were imposed on detrusor strips randomly throughout the SRC or TEA-induced rhythm cycle. The tension amplitude and duration from peak (0%) to peak (100%) of the rhythm cycle were measured immediately prior to each QS, and the NTP tension of the myogenic contraction was measured following each QS (Fig. 3). The duration of the rhythm cycle in which the QS was imposed was assumed to be the same as the previous cycle and used to estimate the percentage of the cycle at which the QS was imposed. Figures 3A and B show tension levels produced by myogenic contractions induced by QS at ∼91% (near the peak) and ∼58% (near the trough), respectively, of the TEA-induced RC cycle.

### QS following pharmacological inhibition of SRC

Previous studies indicate that ROCK activity is required for QS-induced myogenic contraction (Poley et al. 2008), and that both prostaglandins produced by COX isoforms (Collins et al. 2009; Klausner et al. 2011) and ROCK (Ratz and Miner 2003) participate in the regulation of SRC. To test the hypothesis that a common regulatory mechanism is responsible for SRC and QS-induced myogenic contraction, the effects of ROCK and COX inhibitors on each type of contraction were quantified and compared. More specifically, the tension produced by each type of contraction was measured before and during the presence of a ROCK, COX-1, or COX-2 inhibitor, 0.3 *μ*mol/L H-1152, 0.1 *μ*mol/L SC-560 or 0.1 *μ*mol/L NS-398, respectively. SRC amplitude was measured at the end of a 20-min period prior to addition of an inhibitor (control), and at the end of a 20-min exposure to one of the three inhibitors. Average SRC amplitude was calculated as the average amplitude of five consecutive rhythm cycles near the end of each 20-min period. Tissues were also subjected to two QSs at the trough of a SRC cycle, with one at the end of the 20-min period before exposure to the inhibitor and one at the end of the 20-min period in the presence of the inhibitor. As with SRC amplitudes, QS-induced NTP myogenic tension values were measured prior to addition of an inhibitor (control), and during the presence of each inhibitor. The effects of the inhibitors on contraction amplitude values were reported as normalized to the control, predrug amplitude values.

### Statistical analyses

Analyses were performed using Excel (2007; Microsoft, Redmond, WA) or Prism (5.0; GraphPad Software, La Jolla, CA). The *n* value for each experiment refers to the number of animals. To determine significant differences a Student's *t*-test was used when comparing two groups, and when comparing more than two groups, a Student's *t*-test with the Bonferroni correction or a one-way analysis of variance (ANOVA) with the post hoc Student–Newman–Keuls test was used. The null hypothesis was rejected at *P* < 0.05.

## Results

### Effect of QS and rhythm synchronization

**Figure 4:**
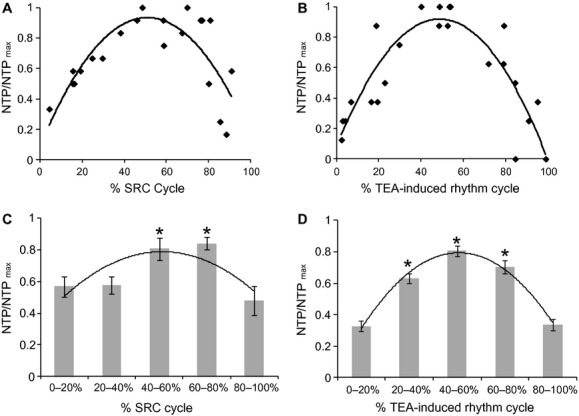
Examples of nadir-to-peak (NTP) myogenic contractions at points throughout spontaneous rhythmic contraction (SRC) (A) and tetraethylammonium (TEA)-induced rhythmic contraction (RC) (B) cycles with second-order polynomial fits (*R*^2^ = 0.63 and 0.75, respectively). Average NTP values for stretches in the ranges of 0–20% 20–40% 40–60% 60–80, and 80–100% of the rhythm cycle (as defined in Fig. 3) for SRC (C) and TEA-induced RC (D) with second-order polynomial fits (*R*^2^ = 0.62 and 0.99, respectively). NTP values for each tissue were normalized to the maximum NTP value for that tissue (NTP_max_). Average NTP values indicated with the * symbol were significantly different from values without the symbol (analysis of variance [ANOVA], *P* < 0.05, *n* = 3 animals, 19–23 stretches per tissue, 9–17 stretches in each cycle range).

NTP tension values for typical detrusor strips were plotted as a function of the percentage of the SRC cycle (Fig. 4A) or the TEA-induced RC cycle (Fig. 4B) at which each QS was initiated, and a second-order polynomial (parabolic) curve was fit to each data set (Fig. 4A–B). Data for all tissues were divided into five groups according to the percentage of the SRC or TEA-induced rhythm cycle at which each QS was imposed (Fig. 4C–D, 0−20, 20−40, 40−60, 60−80, 80−100%), and parabolic curves were fit to each group. Both groups showed parabolic relationships, with tissues in the SRC group producing significantly higher NTP tension in the 40–60, and 60–80% ranges of the rhythm cycle and tissues in the TEA-induced RC group producing higher NTP tension in the 20–40, 40–60, and 60–80% ranges, when the tension amplitude in the RC was relatively low (i.e., during the trough between sequential RCs; Fig. 4C–D).

**Figure 5:**
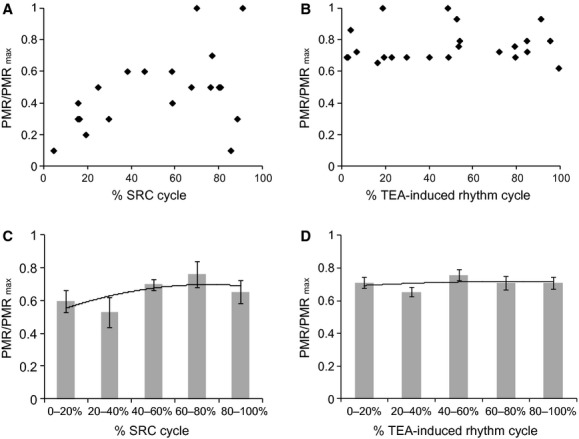
Examples of peak myogenic response (PMR) tension (defined in Fig. 1) at points throughout SRC (A) and tetraethylammonium (TEA)-induced rhythmic contraction (RC) (B) cycles (same detrusor strips as in Fig. 4A–B). PMR values for each tissue were normalized to the maximum PMR value for that tissue (PMR_max_). Average PMR tension for stretches in the ranges of 0–20, 20–40, 40–60, 60–80, and 80–100% of the rhythm cycle (defined in Fig. 3) for SRC (C) and TEA-induced RC (D). Average PMR tension for each of the cycle percentage ranges (0–20, 20–40, 40–60, 60–80, and 80–100%) was not different from that of any of the other cycle ranges for either SRC (C) or TEA-induced rhythm (D) (analyses of variance [ANOVA], *P* > 0.05, *n* = 3 animals, 19–23 stretches per tissue, 9–17 stretches in each cycle range). Second-order polynomial fits (*R*^2^ = 0.45 (C) & 0.08 (D)) produced relatively flat curves compared to the NTP tension data in Fig. 4.

PMR tension values for typical detrusor strips (same strips as in Fig. 4A–B) were also plotted as a function of the percentage of the SRC cycle (Fig. 5A) or the TEA-induced RC cycle (Fig. 5B) at which each QS was initiated (Fig. 5A–B). As with the NTP contraction data, average PMR tension data for all tissues were divided into five groups according to the percentage of the SRC or TEA-induced rhythm cycle at which each QS was imposed (Fig. 4C–D, 0−20, 20−40, 40−60, 60−80, 80−100%). Average PMR tension for each of the cycle percentage ranges (0–20, 20–40, 40–60, 60–80, and 80–100%) was not different from that of any of the other cycle ranges for either SRC (Fig. 5C) or TEA-induced rhythm (Fig. 5D). Thus, PMR tension, in contrast to NTP tension, was independent of the percentage of the rhythm cycle at which the QS was imposed.

### Pharmacological Inhibition of SRC and NTP following QS

**Figure 6:**
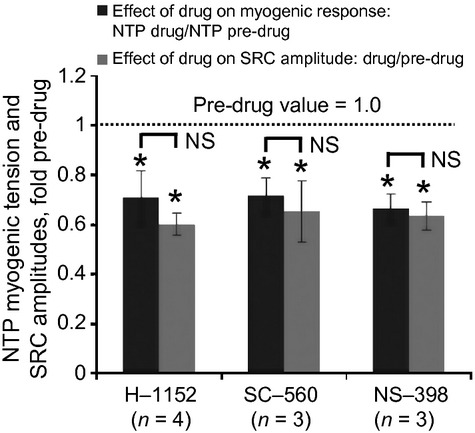
Both nadir-to-peak (NTP) myogenic tension amplitude following quick stretch (QS) and spontaneous rhythmic contraction (SRC) amplitude were inhibited approximately 30–40% by 0.3 *μ*mol/L H-1152, 0.1 *μ*mol/L SC-560, and 0.1 *μ*mol/L NS-398 (NTP and SRC amplitudes were normalized to predrug values, *normalized value < 1.0, analysis of variance (ANOVA), *P* < 0.05, *n* = 3–4). The extent of inhibition was equivalent for both types of phasic contraction (NS, not statistically significant).

The ROCK, COX-1, and COX-2 inhibitors, 0.3 *μ*mol/L H-1152, 0.1 *μ*mol/L SC-560, and 0.1 *μ*mol/L NS-398, respectively, each inhibited NTP myogenic tension following the QS and SRC amplitude by approximately 30–40% (Fig 6, *). Furthermore, H-1152, NS-398, and SC-560 inhibited NTP myogenic tension and SRC amplitude by the same amount (Fig. 6, NS). These data support the hypothesis that SRC and myogenic contraction are regulated by common mechanisms.

## Discussion

### Relationship of myogenic contraction to timing of a QS during the SRC cycle

When focusing on a single twitch contraction from a train of RCs, imposing a QS near the trough between two peaks caused a stronger NTP myogenic contraction than when the QS was imposed near the peak (see Figs. 3 and 4). These data support a model in which a single population of actomyosin crossbridges is responsible for both SRC and stretch-induced myogenic contraction. The NTP tension data suggest that when the mechanism responsible for RC was mostly active (i.e., tension was near a maximum), a smaller proportion of the shared population of crossbridges was available to produce a myogenic contraction in response to a QS. Likewise, we propose that when tension was relatively low (i.e., during the trough between two sequential twitches), the mechanism responsible for inducing RC was largely inactive. Thus, a larger proportion of the shared population of crossbridges was available to produce a strong contraction in response to a QS.

In contrast, PMR tension values were not different for QSs imposed at different points throughout the SRC cycle (see Fig. 5C) or the TEA-induced RC cycle (see Fig. 5D). These data suggest that PMR tension is a measure of the sum of SRC tension and NTP myogenic tension, and that the result of a QS designed to induce a near maximal myogenic contraction was to activate the remaining crossbridges that were not already cycling to produce SRC. Thus, these PMR tension data support the hypothesis that a shared population of crossbridges is responsible for SRC and QS-induced myogenic contraction.

**Figure 7:**
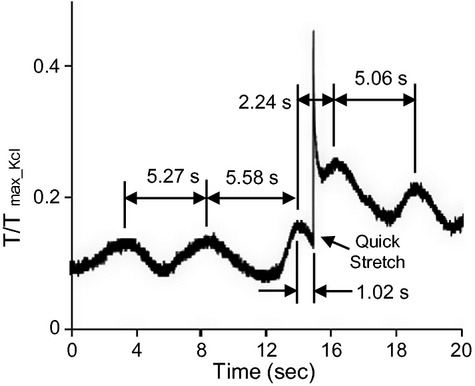
Example of a myogenic contraction due to a quick stretch (QS) imposed 1.02 sec after the peak of a spontaneous rhythmic contraction (SRC) cycle with a period of 5.3–5.6 sec. The peak of the myogenic contraction occurred 2.24 sec after the previous rhythmic peak and 5.06 sec before the next peak, suggesting that the QS shifted the timing of the rhythmic contraction (RC) cycle.

Because the degree of contractile tension is proportional to the number of active crossbridges, the maximum number of active crossbridges during SRC occurs at the peak of each twitch. Likewise, for a given QS stimulus the maximum number of active crossbridges occurs at the peak of the myogenic contraction. If our hypothesis that SRC and QS-induced contraction are due to the regulation of a shared population of crossbridges is valid, this leads to a secondary hypothesis that the QS-induced myogenic contraction in detrusor is the first contraction of a shifted SRC cycle. Data demonstrating stretch-induced RC in rat pulmonary artery (Tanabe et al. 2012) are consistent with this hypothesis. Furthermore, the example in Figure 7 shows a myogenic contraction due to a QS imposed ∼1 sec after the peak of a SRC cycle with a period of ∼5.5 sec. The peak of the myogenic contraction occurred only ∼2.2 sec after the previous rhythmic peak, but ∼5.1 sec before the next peak, suggesting that the QS caused the next peak of the SRC cycle to occur earlier and as a result substantially shifted the timing of the RC cycle with little effect on the SRC frequency. This study was not designed to specifically test this hypothesis, and data from the present set of experiments were insufficient for statistical analysis because tissues were held at the QS length for only 10 sec, which did not provide sufficient time for some tissues to complete a full SRC cycle after the QS. Specifically testing the hypothesis that a QS mechanically resets the SRC cycle in detrusor without altering the frequency will provide additional insight into mechanisms regulating SRC.

In summary, the time within the SRC cycle at which a detrusor strip is subjected to a QS affects the NTP tension (Figs. 4) but not the PMR tension (Figs. 3 and 5) of the resulting myogenic contraction and appears to reset the timing of the SRC cycle (Fig. 7), supporting the hypothesis that SRC and QS-induced contraction are due to a common set of actomyosin crossbridges.

### Regulation of SRC and QS-induced myogenic contraction

ROCK, COX-1, and COX-2 inhibitors affected SRC amplitude and NTP amplitude following a QS to the same degree, providing additional evidence to support the hypothesis that a common regulatory mechanism is responsible for SRC and myogenic contraction due to QS. While the precise mechanism remains to be determined, we previously showed that attenuation of a QS-induced myogenic contraction by a ROCK inhibitor is due to activation of basal myosin phosphatase rather than to inhibition of stretch-activated myosin phosphorylation (Poley et al. 2008). Our studies supported a model, in which RCs can be generated by muscle stretch-induced calcium entry that increases myosin phosphorylation “on top of” a basal level of myosin phosphorylation regulated by ROCK. Rabbit detrusor free of mucosa produces prostaglandins basally (Klausner et al. 2011), and both cyclooxygenases 1 and 2 colocalize with interstitial cells surrounding bundles of detrusor smooth muscle in rabbit (Collins et al. 2009). Moreover, after SRC has been abolished, RC of the same magnitude and frequency can be reestablished by exogenous addition of prostaglandin E_2_ (Collins et al. 2009). Together, these data support a role of prostaglandins in establishing or maintaining rhythmic contractile activity.

As described in the introduction, cell-to-cell synchronization is required to produce the uniform contractions seen during SRC or QS-induced contraction, and because detrusor cells are poorly coupled electrically (Bramich and Brading 1996) and all cells are not innervated (Elbadawi 1995), mechanical coupling is likely responsible for synchronization. Our data showing that a QS-induced contraction mimics a single twitch of an SRC train of contractions support the mechanical coupling model proposed by Elbadawi (1995) and Ji et al. (2002) in which one cell or group of cells rapidly contracts to stretch and stimulate surrounding cells to propagate a contraction throughout a bundle of cells.

### Relevance of a common mechanism for SRC and QS-induced myogenic contraction

The role of stretch-induced contraction in bladder function remains to be determined. Studies of rabbit detrusor indicate that SRC is responsible for regenerating adjustable preload stiffness (APS) (Almasri et al. 2010b) and for length adaptation (Speich et al. 2012b). The present data are consistent with a bladder model that includes two contractile systems, one responsible for producing the voiding contraction, and another responsible for SRC, QS-induced myogenic contraction, generation of APS (Speich et al. 2006, 2012a; Almasri et al. 2010b; Ratz and Speich 2010; Southern et al. 2012), and length adaptation (Speich et al. 2009, 2012b; Almasri et al. 2010a) during filling. These roles are consistent with Gillespie's conclusion that premicturition activity during filling and micturation activity in the bladder are regulated by distinct systems (Gillespie 2004).

Elevated levels of RC have been shown in patients with overactive bladder disorder (Kinder and Mundy 1987; Drake et al. 2005); however, a pathological link between RC and overactive bladder has not been determined. Furthermore, SRC in human and rabbit bladder often has an inconsistent amplitude and/or frequency (Sibley 1984; Byrne et al. 2013) and requires complex analysis to quantify (Byrne et al. 2013; Klausner et al. 2013). If SRC and QS-induced myogenic contraction are due to a common population of actomyosin crossbridges and are regulated by a common mechanism, then QS is a potential mechanical probe to study SRC regulation and its alteration in overactive bladder. More specifically, we propose that a QS protocol could be used to temporally isolate a single SRC twitch or reset the SRC cycle. Thus, QS could be used as a simple alternative or complement to SRC analysis for the comparison of tissues from individuals with and without overactive bladder and during animal testing of agents to specifically target contractile activity during the filling phase.
